# Volumetric analysis of the damage and response of non-invasive brain stimulation in patients with spatial neglect after stroke: a subgroup analysis of the ELETRON trial

**DOI:** 10.1590/1414-431X2025e14800

**Published:** 2025-10-06

**Authors:** M.A. Januzzi, G.J. Luvizutto, L.A. Miranda, T.R. da Silva, F.C. Winckler, S.G.Z. Bazan, T.G.S. Edwards, O.M. Pontes-Neto, R. Bazan, L.E. Betting

**Affiliations:** 1 Departamento de Clínica Médica, Faculdade de Medicina de Botucatu, Botucatu, SP, Brasil; 2 Departamento de Fisioterapia Aplicada, Universidade Federal do Triângulo Mineiro, Uberaba, MG, Brasil; 3 Departamento de Reabilitação, Faculdade de Medicina de Botucatu, Botucatu, SP, Brasil; 4 Departamento de Neurociências e Ciências do Comportamento, Hospital das Clínicas, Faculdade de Medicina de Ribeirão Preto, Universidade de São Paulo, Ribeirão Preto, SP, Brasil; 5 School of Allied Health, The University of Western Australia, Perth, WA, Australia; 6 Departamento de Neurociências e Saúde Mental, Faculdade de Medicina de Botucatu, Botucatu, SP, Brasil

**Keywords:** Stroke, Transcranial direct current stimulation, Hemispatial neglect, Voxel-based lesion mapping, Non-invasive brain stimulation

## Abstract

This study aimed to analyze the compromised cortical and subcortical brain structures and quantify the volume of ischemic lesions in patients with hemispatial neglect after stroke treated with transcranial direct current stimulation (tDCS). This cross-sectional study was conducted using data from the ELETRON Trial. Computed tomography (CT) images of 23 patients who underwent anodal tDCS (A-tDCS), cathodal tDCS (C-tDCS), or placebo (sham-tDCS) were included. Lesion mapping based on high-resolution volumetric CT images was performed using an automated anatomical labeling atlas. The proportion of damage in each region and brain damage between groups were compared using chi-squared and Fisher's exact tests. The behavioral inattention test (BIT-C) score was significantly higher in the C-tDCS group than in sham-tDCS group (P=0.03). Gray matter analysis revealed that lesion extension in the A-tDCS group was 325.580 mm^3^, in C-tDCS was 231.700 mm^3^, and in the sham-tDCS was 241.574 mm^3^. The lesion extension in the white matter was 37.076 mm^3^ in the A-tDCS group, 22.258 mm^3^ in the C-tDCS group, and 40.556 mm^3^ in the sham-tDCS, all centered on the superior longitudinal fasciculus. Overall, the A-tDCS group presented with a larger lesion area in the gray matter than the C-tDCS group (P=0.046). The C-tDCS group showed a smaller proportion of areas with white matter damage than the A-tDCS (P=0.011) and S-tDCS (P=0.002) groups. Hemispatial neglect was significantly improved after C-tDCS; however, the extent of gray and white matter damage was smaller for this group.

## Introduction

Hemispatial neglect is defined as a prevalent perceptual and cognitive impairment following stroke in the right hemisphere (1,2). This condition is a negative predictor of both functional outcomes (3,4) and quality of life (5). Various rehabilitation therapies have been investigated with the aim of mitigating perceptual deficits and improving functional recovery in patients with hemispatial neglect (6-8). Recent clinical trials and meta-analyses have indicated that non-invasive brain stimulation, particularly transcranial direct current stimulation (tDCS) targeted at the right posterior parietal lobe, can reduce the severity of hemispatial neglect after stroke (9-11). Transcranial DCS is a non-invasive stimulation technique that aims to modulate cortical excitability following a stroke. Several studies have explored different methods for improving the efficacy of tDCS in post-stroke rehabilitation protocols (12-14).

Several factors can contribute to the positive or negative responses to tDCS. The positive effects of tDCS on motor recovery depend on the corticospinal tract integrity and baseline motor impairment (13). However, the factors contributing to a positive response to tDCS in perceptual-cognitive recovery after stroke remain unclear. Hemispatial neglect is a complex syndrome involving several cortical and subcortical regions, which is primarily characterized by the disconnection of the right frontoparietal network (15), notably affecting fascicular regions (superior and inferior occipitofrontal and superior longitudinal), basal ganglia, insula, gyri (inferior frontal, superior, and middle temporal), inferior parietal lobe, and operculum (16). Damage to the superior longitudinal and inferior fronto-occipital fasciculi is associated with hemispheric neglect in patients with right hemispheric stroke (17). Disconnection of the posterior portion (splenium) of the corpus callosum has also been shown to increase the severity and persistence of unilateral spatial neglect (USN) following stroke (18).

Volumetric analysis of the damage has revealed that the variability in responses to non-invasive brain stimulation using theta burst transcranial magnetic stimulation (cTBS) is contingent upon the integrity of interhemispheric connections within the corpus callosum, particularly the parieto-parietal connections (19). Typically, patients with intact interhemispheric connectivity demonstrate significant improvement and acceleration in hemispatial neglect recovery and positive functional outcomes following cTBS over the contralesional posterior parietal cortex. However, in the ELETRON Trial clinical study (11), some patients failed to demonstrate satisfactory responses. This raises the question of whether there could be an association between the injured cortical network and response to tDCS in patients with hemispatial neglect after stroke. Indeed, studies have shown that a diminished white matter integrity can negatively affect functional outcomes. Use of tDCS for hemispatial neglect after stroke is not new. However, volumetric analyses and automated anatomical labeling atlas (AAL) in the methods may offer a novel contribution to the literature. Therefore, the present study aimed to examine the compromised cortical and subcortical brain structures, and to quantify the volume of ischemic lesions in patients undergoing tDCS while participating in the ELETRON trial. The present study posited that the extent of lesions in the cortical and/or subcortical pathways may diminish the response to tDCS in patients with hemispheric neglect following a stroke.

## Material and Methods

### Design, setting, and participants

This retrospective study was based on data from the previous multicenter randomized double-blind ELETRON trial (11). The inclusion criteria were patients who completed the ELETRON trial protocol (aged 18 years or older and had suffered hemispatial neglect after ischemic stroke within 6 months of the onset of stroke symptoms) with available high-resolution computed tomography (CT) images. CT images exhibiting technical failures, individuals who did not undergo CT during the clinical trial, or participants who did not complete the protocol were excluded. Ethical approval was obtained from the Botucatu Medical School institutional review board and all participants provided informed consent.

### Procedures

The detailed methods of the ELETRON trial have been described elsewhere (11). In brief, patients were randomly assigned to receive anodal tDCS (A-tDCS), cathodal tDCS (C-tDCS), or a placebo (sham-tDCS), in combination with physical therapy training. For A-tDCS, the anode was placed over the right parietal posterior region (P4), while the cathode was placed over the left supraorbital area (Fp1). For C-tDCS, the cathode was placed over the left parietal posterior region (P3), and the cathode was placed over the right supraorbital area (Fp2). In both the A-tDCS and C-tDCS groups, a constant current of 1 mA was applied for 20 min with a 30-s ramp-up and ramp-down period. For participants in the sham-tDCS group, the stimulator was turned on, but current intensity gradually increased for 30 s, and then tapered off over 30 s. All participants underwent the tDCS protocol for 15 sessions twice a week for a total of 7.5 weeks. Immediately after each tDCS session, all participants underwent 1 h of physical therapy.

The primary outcome of the ELETRON trial was the degree of USN, evaluated by analyzing the differences between the baseline and post-intervention session values of the behavioral inhibition test (BIT-C). The BIT-C is the gold-standard scale used to assess USN in tasks involving drawing and copying figures and line bisection tests. This test comprises six conventional subtests: line cancellation, letter cancellation, star cancellation, line bisection, copying figures and forms, and figure representation. Higher BIT-C scores are associated with fewer perceptual impairments and a smaller degree of hemispatial neglect.

### Lesion mapping and analysis

High-resolution CT was performed during the clinical trial. Only patients with volumetric images were included in the analysis. Imaging was performed using a Toshiba Aquilion (Japan) device with voxels of maximum size 0.46×0.46×1.25 mm. The lesions were semi-automatically segmented using the ITK-SNAP program (20). Subsequently, the images and lesions were registered in the Montreal Neurological Institute standard space using the Clinical Toolbox (21) and SPM8 (Statistical Parametric Mapping, www.fil.ion.ucl.ac.uk/spm/) (22) tools running on the MATLAB^®^ platform (MATrixLABoratory) (23). At this stage, images were transformed into isotropic voxels measuring 1 mm. Finally, overlay maps of the images and locations of the damaged areas were generated using the MRICRON software (24). The lesions were mapped using an automated anatomical labeling atlas (AAL) (25). Gray matter damage analysis in the right hemisphere encompassed a total area of 633.221 mm^3^ with 43 anatomical regions of interest. White matter damage was mapped according to the JHU atlas, which comprises 18 regions of interest and a total area of 85.891 mm^3^.

### Statistical analysis

Data normality was assessed using the Shapiro-Wilk test. Comparisons of clinical and demographic data among the groups were conducted using the Kruskal-Wallis' test (Dunn *post hoc* test) for continuous variables and Fisher's exact test for categorical data. Comparison of the proportion of damage in each region of interest and brain damage among the groups was performed using chi-squared and Fisher's exact tests. Statistical significance was set at P<0.05, with Bonferroni correction applied for multiple comparisons. SPSS version 25.0 (IBM Corp., USA) software was used for all statistical analyses.

## Results

Of the 46 patients included in the ELETRON trial, 23 were excluded due to poor image quality or technical processing failure. Subsequently, a total of 23 patients were included in the lesion mapping imaging study and divided into the A-tDCS (n=8), C-tDCS (n=8), and sham-tDCS (n=7) groups. The clinical and demographic data of the participants included in the study are presented in Table 1. No significant differences were observed in clinical or demographic data between the groups. No significant differences were observed in BIT scores between groups at baseline (F=8.5; P=0.09); however, post-intervention scores showed a significant difference (F=14.7; P=0.02), with *post hoc* analysis showing a difference between the C-tDCS and sham groups (mean difference [MD], 29.8; P=0.03).

**Table 1:** Clinical and demographic data of the three groups.

	A-tDCS (n=8)	C-tDCS (n=8)	Sham-tDCS (n=7)	P
Demographic variables				
Age (year)^1^	67.0 (40.0-80.0)	66.0 (42.0-84.0)	65.5 (35.0-80.0)	0.26
Female sex n, (%)^2^	5 (62.5)	4 (50.0)	4 (57.1)	0.88
White race n, (%)^2^	4 (50.0)	4 (50.0)	3 (42.8)	0.95
Medical history n, (%)				
Prior stroke/TIA n, (%)^2^	2 (25.0)	2 (25.0)	2 (28.5)	0.98
Diabetes mellitus n, (%)^2^	5 (62.5)	5 (33.3)	5 (71.4)	0.92
Hypertension n, (%)^2^	7 (87.5)	6 (80.0)	6 (85.7)	0.78
Currents or past tobacco use n, (%)^2^	6 (75.0)	5 (53.3)	4 (57.1)	0.75
Obesity n, (%)^2^	2 (25.0)	2 (25.0)	1 (14.3)	0.84
Heart disease n, (%)^2^	2 (25.0)	2 (25.0)	1 (14.3)	0.84
Hospitalization				
Thrombolysis n, (%)^2^	2 (25.0)	2 (25.0)	2 (28.5)	0.98
Time of hospitalization (days)^1^	9.0 (1.0-20.0)	9.0 (2.0-15.0)	9.0 (1.0-15.0)	0.86
NIHSS (hospital discharge)^1^	6.0 (4.0-15.0)	4.0 (1.0-14.0)	4.0 (0.0-15.0)	0.67
mRS (hospital discharge)^1^	3.0 (1.0-4.0)	3.0 (1.0-4.0)	3.0 (1.0-5.0)	0.88
Time after stroke (days)^1^	45.0 (15.0-60.0)	42.0 (10.0-55.0)	40.0 (17.0-70.0)	0.52
Body impairments				
Right hemiplegia n, (%)^2^	8 (100.0)	8 (100.0)	8 (100.0)	1.00
Anosognosia n, (%)^2^	2 (25.0)	0 (0.0)	0 (0.0)	0.11
Pusher syndrome n, (%)^2^	1 (12.5)	0 (0.0)	1 (12.5)	0.58
BIT-C				
Baseline^1^	80.5 (46-116)	106.3 (30-126)	95.3 (44-125)	0.35
After intervention^1^	119.8 (80-139)	129.1 (94-146)	99.3 (53-124)	0.002

Data are reported as median and interquartile interval. ^1^ANOVA; ^2^chi-squared test. A-tDCS: anodal transcranial direct current stimulation: C-tDCS: cathodal tDCS; TIA: transient ischemic attack; NIHSS: National Institutes of Health Stroke Scale; mRS: modified Rankin scale; BIT-C: Behavioral Inattention Test (conventional).

**Figure 1:**
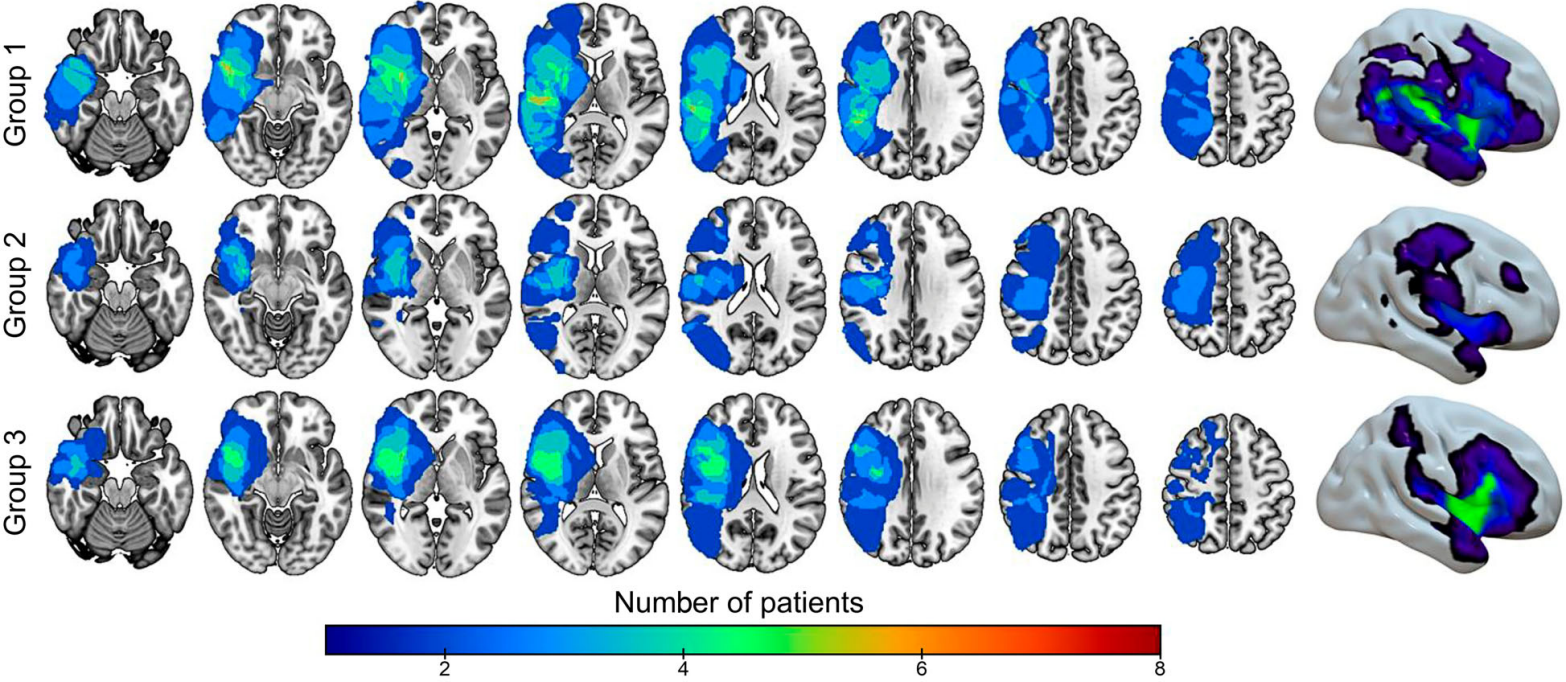
Overlap of the lesion maps for each group investigated group using an axial magnetic resonance imaging template and 3D rendering. The color scale indicates the number of affected individuals. Group 1: anodal transcranial direct current stimulation (A-tDCS); Group 2: cathodal tDCS (C-tDCS); Group 3: Sham-tDCS.

Figure 1 shows the total extent of the lesions in each group. In the A-tDCS group, the lesion extension in the gray matter was 325.580 mm^3^ (51% of all regions of interest [ROIs]), primarily centered in the opercular Rolandic area (x=42, y=-13, z=17) and involving all 43 ROIs (100%). In the C-tDCS group, the lesion extension in the gray matter was 231.700 mm^3^ (37% of all ROIs), centered on the amygdala (x=38, y=-10, z=6), and involved 37/43 ROIs (86%). In the sham-tDCS group, the lesion extension was 241.574 mm^3^ (38% of all ROIs), centered on the insula (x=37, y=-7, z=17), and involved 41/43 ROIs (95%).

In the A-tDCS group, the lesion extension in the white matter was 37.076 mm^3^ (43% of all ROIs), centered on the superior longitudinal fasciculus (SLF) (x=42, y=-13, z=17), and involved 18/18 regions of interest (100%). In the C-tDCS group, the lesion extension in the white matter was 22.258 mm^3^ (26% of all ROIs), centered on the SLF (x=38, y=-10, z=25), and involved 15/18 regions of interest (83%). In the sham-tDCS group, the lesion extension in the white matter was 40.556 mm^3^ (47% of all ROIs), centered on the SLF (x=37, y=-7, z=17), and involved 18/18 ROIs (100%).

Tables 2 and 3 show the brain regions of interest, total number of voxels, number of voxels with lesions, and equivalent percentages for each group. In general, the A-tDCS group exhibited a larger lesion area in the gray matter than the C-tDCS group (P=0.046). Furthermore, the C-tDCS group demonstrated a lower proportion of areas with white matter damage than the A-tDCS (P=0.011) and sham-tDCS (P=0.002) groups. Regarding the overall injured brain area (gray and white matter), the A-tDCS group measured 362.656 mm^3^ (50%), the C-tDCS group measured 253.958 mm^3^ (35%), and the sham-tDCS group measured 282.130 mm^3^ (39%), with statistically significant differences between the A-tDCS and C-tDCS groups (P=0.03).

**Table 2:** Overview of the gray matter regions of interest showing the total number of voxels, the number of voxels with lesions, and the equivalent percentage for each group.

Brain region		A-tDCS (1)		C-tDCS (2)		Sham-tDCS (3)		P value (*post hoc*)		
	Total voxels	Voxel with lesion	% lesion	Voxel with lesion	% lesion	Voxel with lesion	% lesion	1 *vs* 2	2 *vs* 3	1 *vs* 3
Precentral gyrus	27058	16592	61	17218	64	10122	37	0.37	0.013	0.08
Superior frontal gyrus	32089	9537	30	15114	47	7801	24	0.39	0.079	0.29
Superior frontal gyrus (pars orbitaris)	7859	957	12	48	1	1672	21	0.13	<0.0001	0.44
Middle frontal gyrus	40374	30380	75	26763	66	14561	36	0.33	<0.0001	<0.0001
Middle frontal gyrus (pars orbitaris)	8057	2129	26	391	5	2211	27	<0.0001	<0.0001	0.09
Inferior frontal gyrus (pars opercularis)	11174	11170	100	5107	46	10453	94	<0.0001	<0.0001	0.08
Inferior frontal gyrus (pars triangularis)	17132	17131	100	15328	89	14857	87	<0.0001	0.08	0.013
Inferior frontal gyrus (pars orbitaris)	13747	9092	66	8891	65	11275	82	0.55	0.79	0.23
Rolandic operculum	10733	10649	99	8360	78	10184	95	<0.0001	<0.0001	0.07
Supplementary motor area	18885	218	1	2078	11	4934	26	0.26	0.79	<0.0001
Olfactory	2286	163	7	163	7	599	26	0.13	0.026	0.026
Frontal superior medial	16979	1011	6	58	0	612	4	NS	NS	NS
Rectus	5930	121	2	0	0	550	9	NS	0.264	NS
Insula	14128	14037	99	14009	99	14128	100	0.89	0.92	0.88
Hippocampus	7606	5995	79	1914	25	3445	45	<0.0001	<0.0001	<0.0001
Parahippocampal gyrus	9028	857	9	268	3	1521	17	NS	0.1188	NS
Amygdala	1965	1716	87	721	37	1908	97	<0.0001	<0.0001	NS
Calcarine gyrus	14885	423	3	0	0	0	0	NS	NS	NS
Cuneus	11323	375	3	0	0	0	0	NS	NS	NS
Lingual	18450	124	1	0	0	0	0	NS	NS	NS
Superior occipital gyrus	11149	1820	16	1224	11	834	7	NS	NS	NS
Middle occipital gyrus	16512	10209	62	7101	43	5826	35	0.079	NS	<0.0001
Inferior occipital gyrus	7929	942	12	0	0	0	0	0.039	NS	0.039
Fusiform	20227	2300	11	488	2	548	3	NS	NS	NS
Postcentral gyrus	30652	20221	66	24665	80	13584	44	NS	<0.0001	0.13
Superior parietal lobule	17554	7357	42	7175	41	4216	24	NS	NS	0.79
Inferior parietal lobule	10763	10763	100	6011	56	9054	84	<0.0001	<0.0001	<0.0001
Supra marginal	15770	15754	100	10024	64	12415	79	<0.0001	NS	<0.0001
Angular	14009	13416	96	4827	34	13010	93	<0.0001	<0.0001	NS
Precuneus	26083	1311	5	857	3	162	1	0.92	0.96	0.79
Paracentral lobule	6693	106	2	95	1	548	8	0.89	0.66	0.72
Caudate	7941	4443	56	1335	17	7547	95	<0.0001	<0.0001	<0.0001
Putamen	8510	8066	95	6504	76	8510	100	0.0132	<0.0001	0.69
Pallidum	2188	2035	93	1353	62	2188	100	<0.0001	<0.0001	0.81
Thalamus	8399	2994	36	804	10	2362	28	<0.0001	0.13	0.82
Heschl	1936	1930	100	1773	92	1917	99	0.39	NS	NS
Superior temporal gyrus	25258	24612	97	15816	63	16643	66	<0.0001	NS	<0.0001
Superior temporal polar gyrus	10654	7688	72	6645	62	8403	79	0.82	0.79	0.93
Middle temporal gyrus	35484	34511	97	14847	42	13448	38	<0.0001	0.08	<0.0001
Middle temporal polar gyrus	9470	3761	40	2435	26	5526	58	0.28	<0.0001	0.11
Inferior temporal gyrus	28468	18402	65	560	2	3835	13	<0.0001	0.39	<0.0001
Total	633221	325580	51	231700	37	241574	38	0.046	0.88	0.06

ANOVA and Bonferroni correction for multiple comparisons; NS: non-significant. A-tDCS: anodal transcranial direct current stimulation: C-tDCS: cathodal tDCS.

**Table 3:** Overview of the white matter regions of interest and the total number of voxels, the number of voxels with lesions, and the equivalent percentage for each group.

Brain region		A-tDCS (1)		C-tDCS (2)		Sham-tDCS (3)		P value (*post hoc*)		
	Total voxels	Voxel withlesion	% lesion	Voxel withlesion	% lesion	Voxel withlesion	% lesion	1 *vs* 2	2 *vs* 3	1 *vs* 3
Corpus callosum (genus)	8851	152	2	0	0	1532	17	NS	<0.0001	0.01
Corpus callosum (body)	13711	548	4	15	0	2444	18	NS	NS	0.057
Corpus callosum (splenium)	12729	8	0	0	0	10	0	NS	NS	NS
Internal capsule (anterior limb)	3018	2643	88	1079	36	3012	100	<0.0001	<0.0001	0.01
Internal capsule (posterior limb)	3752	3275	87	1580	42	2906	77	<0.0001	<0.0001	NS
Internal capsule (retrolentiform)	2469	2053	83	990	40	1824	74	<0.0001	<0.0001	NS
Corona radiata (anterior)	6852	4725	69	540	8	5430	79	<0.0001	<0.0001	NS
Corona radiata (superior)	7508	5077	68	5492	73	7179	96	NS	0.0003	<0.0001
Corona radiata (posterior)	3714	987	27	420	11	1950	53	0.22	<0.0001	0.005
Posterior thalamic radiation	3978	2369	60	654	16	137	3	<0.0001	0.09	<0.0001
Sagittal stratum^*^	2231	1964	88	598	27	942	42	<0.0001	NS	<0.0001
External capsule	5587	5429	97	4806	86	5587	100	0.28	0.005	NS
Fornix	1125	939	83	286	25	328	29	<0.0001	NS	<0.0001
Superior longitudinal fasciculus	6605	5807	88	5209	79	6380	97	NS	0.005	NS
Superior fronto-occipital fasciculus	507	507	100	224	44	507	100	<0.0001	<0.0001	NS
Tapetum	600	74	12	0	0	9	2	0.017	NS	0.31
Total	85891	37076	43	22258	26	40556	47	0.011	0.002	0.56

*Includes the inferior longitudinal and inferior frontooccipital fasciculi; ANVOA and Bonferroni correction for multiple comparisons; NS: non-significant. A-tDCS: anodal transcranial direct current stimulation: C-tDCS: cathodal tDCS.

## Discussion

The present study mapped the ischemic areas of the gray and white matter in a subset of patients from the ELETRON trial (11). In this subset, larger lesion areas were observed in the A-tDCS group. The C-tDCS group further demonstrated a superior improvement in BIT-C scores compared to the sham group. The white matter damage observed in all three groups was centered on the SLF, which was particularly prominent in the A-tDCS group. In terms of gray matter damage, the A-tDCS group exhibited damage centered in the opercular Rolandic area, the C-tDCS had damage in the amygdala, and the sham-tDCS in the insula. However, the overall extent of gray and white matter damage was smaller in the C-tDCS group.

Stroke affecting the non-dominant hemisphere, typically the right hemisphere, particularly fascicular regions (superior and inferior occipitofrontal and superior longitudinal), basal ganglia, insula, inferior frontal gyrus, superior temporal gyrus, middle temporal gyrus, inferior parietal lobe, and operculum, is associated with attention and visuospatial dysfunction (17,26,27). In our analysis of gray matter regions, the sham group exhibited a greater number of lesions centered on the insula, a region involved in somatosensory and auditory processing, and connected to the inferior parietal and lateral prefrontal cortices (26). Insular lesions may manifest as non-visual hemispatial neglect symptoms, such as impairment of body exploration or tactile extinction (27). The A-tDCS group displayed a larger lesion centered on the Rolandic operculum. Several studies have linked the rolandic operculum to emotional processing, with high-degree lesions in this region being associated with apathy, depression, and anxiety following a stroke (28). Additionally, lesions in the rolandic operculum are associated with anosognosia following a stroke (29), resulting in multiple concomitant deficits in sensation, attention, interoceptive bodily representations, motor programming, error monitoring, memory, and affective processing (30). The C-tDCS group exhibited greater damage to the amygdala than the control group. The amygdala plays a fundamental role in emotional processing (31), and has an attentional network capable of enhancing perceptual processing, potentially facilitating the conscious perception of undetected stimuli (32).

In the analysis of the white matter regions, all groups exhibited lesions in the SLF, albeit to a lesser extent in the C-tDCS group, which showed a better therapeutic response in reducing hemispheric neglect following stroke. The SLF links the superior parietal region and the adjacent medial parietal cortex with the supplementary and premotor areas in the frontal lobe (33). Functional MRI studies have previously demonstrated coactivation of the parietal and frontal areas when performing a broad range of visual-spatial tasks (34). Damage to the parietofrontal projections is the strongest predictive factor for chronic spatial neglect following a stroke (35). Similar to the association between cortico-spinal tract integrity and improved motor recovery, as well as the therapeutic effects of post-stroke tDCS (13), this study underscores the importance of white matter integrity, particularly in the SLF, for perceptual recovery and selective indication of tDCS in patients with hemispatial neglect.

The ELETRON trial revealed the superiority of A-tDCS over sham treatment in terms of BIT-C (11). However, our subgroup analysis, which excluded patients due to technical failures in imaging, revealed a larger lesion area in both the gray and white matter in the A-tDCS group. However, this benefit was not sustained, suggesting that the efficacy of tDCS may be more pronounced in patients with gray and white matter integrity. Looking at the maxim “A car may start, but without a fit driver or a clear road, it won't reach its destination”, the car represents tDCS, the driver represents the qualified therapist, and the road represents intact cortical and subcortical pathways for conducting the stimulus.

This study had some limitations. First, this was a subgroup of patients included in a previous randomized clinical trial. Therefore, the results have reduced statistical power due to loss of patients. Second, the sample size was reduced because of technical image limitations such as segmentation errors, inadequate image quality, hemorrhage, microangiopathy, and unsatisfactory resolution. The main issue encountered was that for some patients, the boundary between recent and previous lesions was unclear. In such cases, the segmentation algorithm fails and is unable to accurately classify the damaged tissue. This problem may be challenging to address even with visual analysis using CT scans. Third, the use of CT rather than magnetic resonance imaging may have limited anatomical precision. Despite these limitations, this study offers valuable insights into the factors influencing individual responses to tDCS. Future studies should individualize the location of brain stimulation based on neuroimaging studies, while patient selection for tDCS should be explored in large-scale phase III clinical trials.

## Conclusion

The extent of white matter damage was centered on the SLF in all three groups. Individuals in the A-tDCS group exhibited gray matter damage centered on the opercular Rolandic area, whereas those in the C-tDCS group exhibited gray matter damage centered on the amygdala. However, the overall extent of gray and white matter damage was lower in the C-tDCS group. In this sub-study, patients in the C-tDCS group showed significant improvement compared with those in the sham-tDCS group, an effect which was attributable to the smaller extent of damage in the white matter regions.

## References

[1] Ringman JM, Saver JL, Woolson RF, Clarke WR, Adams HP (2004). Frequency, risk factors, anatomy, and course of unilateral neglect in an acute stroke cohort. Neurology.

[2] Corbetta M, Shulman GL (2011). Spatial neglect and attention networks. Annu Rev Neurosci.

[3] Nijboer TCW, Kollen BJ, Kwakkel G (2014). The impact of recovery of visuo-spatial neglect on motor recovery of the upper paretic limb after stroke. Plos One.

[4] Luvizutto GJ, Moliga AF, Rizzatti GRS, Fogaroli MO, de Moura E, Nunes HRC, Resense LAL (2018). Unilateral spatial neglect in the acute phase of ischemic stroke can predict long-term disability and functional capacity. Clinics (Sao Paulo).

[5] Sobrinho KRF, Santini ACM, Marques CLS, Gabriel MG, de Moura E, de Souza LAPS (2018). Impact of unilateral spatial neglect on chronic patient's post-stroke quality of life. Somatosens Mot Res Sep.

[6] Bowen A, Hazelton C, Pollock A, Lincoln NB (2013). Cognitive rehabilitation for spatial neglect following stroke. Cochrane Database Syst Rev.

[B07] Azouvi P, Jacquin-Courtois S, Luauté J (2017). Rehabilitation of unilateral neglect: Evidence-based medicine. Ann Phys Rehabil Med.

[8] Hazelton C, Thomson K, Todhunter-Brown A, Campbel P, Chung CS, Dorris L (2022). Interventions for perceptual disorders following stroke. Cochrane Database Syst Rev.

[9] Kashiwagi FT, El Dib R, Gomaa H, Gawish N, Suzumura EA, da Silva TR (2018). Noninvasive brain stimulations for unilateral spatial neglect after stroke: a systematic review and meta-analysis of randomized and nonrandomized controlled trials. Neural Plast.

[B10] Salazar APS, Vaz PG, Marchese RR, Stein C, Pinto C, Pagnussat AS (2018). Noninvasive brain stimulation improves hemispatial neglect after stroke: systematic review and meta-analysis. Arch Phys Med Rehabil.

[11] da Silva TR, Nunes HRC, Martins LG, da Costa RDM, de Souza JT, Winckler FC (2022). Non-invasive brain stimulation can reduce unilateral spatial neglect after stroke: ELETRON trial. Ann Neurol.

[12] Stockbridge MD, Elm J, Teklehaimanot AA, Cassarly C, Spell LA, Fridriksson, et al (2023). Individual differences in response to transcranial direct current stimulation with language therapy in subacute stroke. Neurorehabil Neural Repair.

[B13] Van Hoornweder S, Vanderzande L, Bloemers E, Verstraelen S, Depestele S, Cuypers K (2021). The effects of transcranial direct current stimulation on upper-limb function post-stroke: a meta-analysis of multiple-session studies. Clin Neurophysiol.

[14] Chhatbar PY, Ramakrishnan V, Kautz S, George MS, Adams RJ, Feng W (2016). Transcranial direct current stimulation post-stroke upper extremity motor recovery studies exhibit a dose-response relationship. Brain Stimul.

[15] Bird CM, Malhotra P, Parton A, Coulthard E, Rushworth MFS, Husain M (2006). Visual neglect after right posterior cerebral artery infarction. J Neurol Neurosurg Psychiatry.

[16] Palmerini F, Bogousslavsky J (2012). Right hemisphere syndromes. Front Neurol Neurosci.

[17] Kwon BM, Lee JY, Ko N, Kim BR, Moon WJ, Choi DH (2022). Correlation of hemispatial neglect with white matter tract integrity: A DTI study. Brain Neurorehabil.

[18] Bozzali M, Mastropasqua C, Cercignani M, Giulietti G, Bonni S, Caltagirone C (2012). Microstructural damage of the posterior corpus callosum contributes to the clinical severity of neglect. PloS One.

[19] Nyffeler T, Vanbellingen T, Kaufmann BC, Pflugshaupt T, Bauer D, Frey J (2019). Theta burst stimulation in neglect after stroke: Functional outcome and response variability origins. Brain.

[20] Yushkevich PA, Piven J, Hazlett HC, Smith RG, Ho S, Gee JC (2006). User-guided 3d active contour segmentation of anatomical structures: significantly improved efficiency and reliability. Neuroimage.

[21] Rorden C, Bonilha L, Fridriksson J, Bender B, Karnath HO (2012). Age-specific ct and mri templates for spatial normalization. Neuroimage.

[22] Friston KJ, Ashburner JT, Kiebel SJ, Nichols TE, Penny WD (2006). Statistical parametric mapping: the analysis of functional brain images.

[23] MATLAB (2012). Statistics toolbox release. natick, Massachusetts.

[24] Rorden C, Brett M (2000). Stereotaxic display of brain lesions. Behav Neurol.

[25] Tzourio-Mazoyer N, Landeau B, Papathanassiou D, Crivello F, Etard O, Delcroix N (2002). Automated anatomical labeling of activations in SPM using a macroscopic anatomical parcellation of the mni mri single-subject brain. Neuroimage.

[26] Augustine JR (1996). Circuitry and functional aspects of the insular lobe in primates including humans. Brain Res Brain Res Rev.

[27] Manes F, Paradiso S, Springer JA, Lamberty G, Robinson RG (1999). Neglect after right insular cortex infarction. Stroke.

[28] Sutoko S, Atsumori H, Obata A, Funane T, Kandori A, Shimonaga K (2020). Lesions in the right Rolandic operculum are associated with self-rating affective and apathetic depressive symptoms for post-stroke patients. Sci Rep.

[29] Husain M, Kennard C (1996). Visual neglect associated with frontal lobe infarction. J Neurol.

[30] Vocat R, Staub F, Stroppini T, Vuilleumier P (2010). Anosognosia for hemiplegia: a clinical-anatomical prospective study. Brain.

[31] Russell C, Li K, Malhotra PA (2013). Harnessing motivation to alleviate neglect. Front Hum Neurosci.

[32] Grabowska A, Marchewka A, Seniów J, Polanowska K, Jednoróg K, Królicki L (2011). Emotionally negative stimuli can overcome attentional deficits in patients with visuo-spatial hemineglect. Neuropsychologia.

[33] Doricchi F, Thiebaut de Schotten M, Tomaiuolo F, Bartolomeo P (2008). White matter (dis)connections and gray matter (dys)functions in visual neglect: Gaining insights into the brainNetworks of spatial awareness. Cortex.

[34] Husain M, Nachev P (2007). Space and the parietal cortex. Trends Cogn Sci.

[35] Golay L, Schnider A, Ptak R (2008). Cortical and subcortical anatomy of chronic spatial neglect following vascular damage. Behav Brain Funct.

